# Mapping and Validation of *qHD7b:* Major Heading-Date QTL Functions Mainly under Long-Day Conditions

**DOI:** 10.3390/plants11172288

**Published:** 2022-09-01

**Authors:** Amir Sohail, Liaqat Shah, Ling Liu, Anowerul Islam, Zhengfu Yang, Qinqin Yang, Galal Bakr Anis, Peng Xu, Riaz Muhammad Khan, Jiaxin Li, Xihong Shen, Shihua Cheng, Liyong Cao, Yingxin Zhang, Weixun Wu

**Affiliations:** 1 State Key Laboratory of Rice Biology, China National Rice Research Institute, Hangzhou 310006, China; 2 Department of Agriculture, Mir Chakar Khan Rind University, Sibi 82000, Pakistan; 3 Department of Agricultural Extension, Ministry of Agriculture, Dhaka 1215, Bangladesh; 4 State Key Laboratory of Subtropical Silviculture, Zhejiang A&F University, Hangzhou 311300, China; 5 Rice Research and Training Center, Field Crops Research Institute, Agriculture Research Center, Kafrelsheikh 33717, Egypt; 6 Cereal Crops Research Institute (CCRI) Pirsabak Nowshera, Agriculture Research System, Nowshera 24100, Pakistan; 7 Northern Center of China National Rice Research Institute, Shuangyashan 155600, China

**Keywords:** rice (*Oryza sativa* L.), quantitative trait locus, chromosome segment substitution lines, *qHD7b*, fine-mapping

## Abstract

Heading date (HD) is one of the agronomic traits that influence maturity, regional adaptability, and grain yield. The present study was a follow-up of a previous quantitative trait loci (QTL) mapping study conducted on three populations, which uncovered a total of 62 QTLs associated with 10 agronomic traits. Two of the QTLs for HD on chromosome 7 (*qHD7a* and *qHD7b*) had a common flanking marker (RM3670) that may be due to tight linkage, and/or weakness of the statistical method. The objectives of the present study were to map QTLs associated with HD in a set of 76 chromosome segment substitution lines (CSSLs), fine map and validate one of the QTLs (*qHD7b*) using 2997 BC_5_F_2:3_ plants, and identify candidate genes using sequencing and expression analysis. Using the CSSLs genotyped with 120 markers and evaluated under two short-day and two long-day growing conditions, we uncovered a total of fourteen QTLs (*qHD2a*, *qHD4a*, *qHD4b*, *qHD5a*, *qHD6a*, *qHD6b*, *qHD7b*, *qHD7c*, *qHD8a*, *qHD10a*, *qHD10b*, *qHD11a*, *qHD12a*, and *qHD12b*). However, only *qHD6a* and *qHD7b* were consistently detected in all four environments. The phenotypic variance explained by *qHD6a* and *qHD7b* varied from 10.1% to 36.1% (mean 23.1%) and from 8.1% to 32.8% (mean 20.5%), respectively. One of the CSSL lines (CSSL52), which harbored a segment from the early heading XieqingzaoB (XQZB) parent at the *qHD7b* locus, was then used to develop a BC_5_F_2:3_ population for fine mapping and validation. Using a backcross population evaluated for four seasons under different day lengths and temperatures, the *qHD7b* interval was delimited to a 912.7-kb region, which is located between RM5436 and RM5499. Sequencing and expression analysis revealed a total of 29 candidate genes, of which *Ghd7* (*Os07g0261200*) is a well-known gene that affects heading date, plant height, and grain yield in rice. The *ghd7* mutants generated through CRISPR/Cas9 gene editing exhibited early heading. Taken together, the results from both the previous and present study revealed a consistent QTL for heading date on chromosome 7, which coincided not only with the physical position of a known gene, but also with two major effect QTLs that controlled the stigma exertion rate and the number of spikelets in rice. The results provide contributions to the broader adaptability of marker-assisted breeding to develop high-yield rice varieties.

## 1. Introduction

Rice is a staple food for more than 50% of the world’s population, with its production expected to increase by about 25% in 2030 to keep pace with population growth. Rice is a facultative, short-day crop that flowers earlier under short-day (SD) conditions and later under long-day (LD) conditions [1]. Heading date (HD) is a crucial trait affecting rice adaption to diverse cultivation areas, cropping seasons, maturity, and grain yield [2]. The development of early- or late-maturing cultivars depends on ecological conditions. In the regions where growing seasons are short, the aim is to develop early maturing varieties to escape frost damage, but there may be a yield penalty. However, in the regions where growing seasons are long, the aim is to develop late-maturing varieties with all of the assimilates efficiently transmitted to the grains, thereby enhancing grain weight and yield. Generally, there was a trade-off between flowering time and yield, which aimed to maximize production [3].

Numerous studies have been conducted to map and characterize 712 HD genes and quantitative trait loci (QTLs) that have been documented in the Gramene database (http://archive.gramene.org/qtl/ (accessed on 29 August 2022)). The *E1/Ghd7* was the first HD QTL reported in rice, which possesses a functional dominant *E1* allele and a non-functional *e1* allele [4]. The allelic variation of *Ghd7* contributes to the geographic distribution of cultivated rice [5], which has been investigated for photoperiod sensitivity and regional adaptability [6]. The functional *Ghd7* alleles (e.g., *Ghd7-1*, *Ghd7-2*, and *Ghd7-3*) delay heading, while the non-functional alleles (e.g., *Ghd7-0* and *Ghd7-0a*) shorten heading date in the different genetic backgrounds of rice. Both the *Ghd7-1* and *Ghd7-3* alleles were found in rice varieties grown in the tropics, subtropics, and areas with hot summers and long growing seasons in China and Southeast Asia. The *Ghd7-2* allele was found in temperate *japonica* varieties from Japan and northern China and had a smaller phenotypic effect than *Ghd7-1* [7]. *Se1*/*Hd1* was the first cloned heading-date QTL, an ortholog of *Arabidopsis CONSTANS* that promotes and suppresses flowering under short- and long-day growing conditions, respectively [8]. *Heading date 6* (*Hd6*) [9], *Heading date 3a* (*Hd3a*) [10], *Early heading date 1* (*Ehd1*) [11], *Days to heading 8* (*DTH8*)/*Ghd8* [12], *Heading date 17* (*Hd17*) [13], *RICE FLOWERING LOCUS T 1* (*RFT1*) [14], and *Days to heading 2* (*DTH2*) [3] are other HD QTLs in rice that have been cloned using a map-based approach. The analysis of these genes exhibited two main photoperiodic flowering pathways in rice: *Hd1-Hd3a* and *Ghd7-Ehd1-Hd3a*/*RFT1*. Major QTLs associated with the late heading, such as *Ghd7*, *Hd1*, *DTH8*/*Ghd8*, and *DTH7*/*Ghd7.1* [5,15], showed a strong correlation with an increase in grain yield, which suggests that the use of such types of HD QTLs can significantly influence rice’s productivity and adaptability to specific growing conditions. 

Recent progress in molecular technology and statistical methodology has provided researchers an opportunity to map and characterize HD QTLs in diverse types of populations, including F_2_, recombinant inbred lines (RILs), and doubled haploid lines (DHLs) in rice [16,17]. However, these populations may not be ideal for the precise mapping of QTLs due to the simultaneous segregation of multiple loci originating from the two parents. Moreover, it would be more challenging to determine the actual genetic actions of the QTLs and differentiate the QTL effects from background noise [18,19]. Chromosome segment substitution lines (CSSLs) are genetic stocks that consist of overlapping segments of the complete genome of any genotype. CSSLs have been widely used to map QTLs accurately, evaluate gene interactions, discover new alleles, and compare the phenotypic effect of genes or QTLs [19,20]. Further fine mapping of QTLs of interest can be done by constructing segregating populations obtained from crossing one of the CSSLs and their recurrent parent [21].

In a previous study, our group identified 9 HD QTLs in a RIL population (Figure S1) and 2 BCF_1_ populations derived from a cross between XieqingzaoB (XQZB) and Zhonghui9308 (ZH9308), which individually accounted for 2.6–18.6% of the phenotypic variance [22]. Three of the nine HD QTLs were mapped on chromosome 7 between RM3670 and RM2 markers (*qHD7a*), between RM5436 and RM3670 (*qHD7b*), and between RM118 and RM3555 (*qHD7c*), explaining 18.6%, 12.1%, and 5.6%, respectively. The *qHD7a* and the *qHD7b* QTL were physically located between 13,439,924–16,022,676 bp and 9,075,636–1,343,9924 bp, respectively. RM3670, located at 13,439,924 bp, was a common flanking marker in both *qHD7a* and *qHD7b*, suggesting that the two QTLs are either tightly linked or the statistical method wrongly identified them as two independent QTLs. Both issues may be resolved using CSSLs, which form the basis of the present study. Therefore, the objectives of the present study were to understand the phenotypic variation of the CSSLs for heading date, fine map the HD QTL on chromosome 7, and identify candidate genes associated with HD under short- and long-day rice-growing conditions. Furthermore, we were also interested in determining the proportion of phenotypic variance explained by one of the QTLs on chromosome 7, validating and fine mapping its position using the BC_5_F_2:3_ population derived from a cross between one of the CSSLs and the recurrent parent, identifying candidate genes near the target QTL, and determining its actual effect in mutants generated through CRISPR/Cas9 gene editing. 

## 2. Results

### 2.1. Phenotypic and Genotypic Analysis of CSSLs

**Figure 1:**
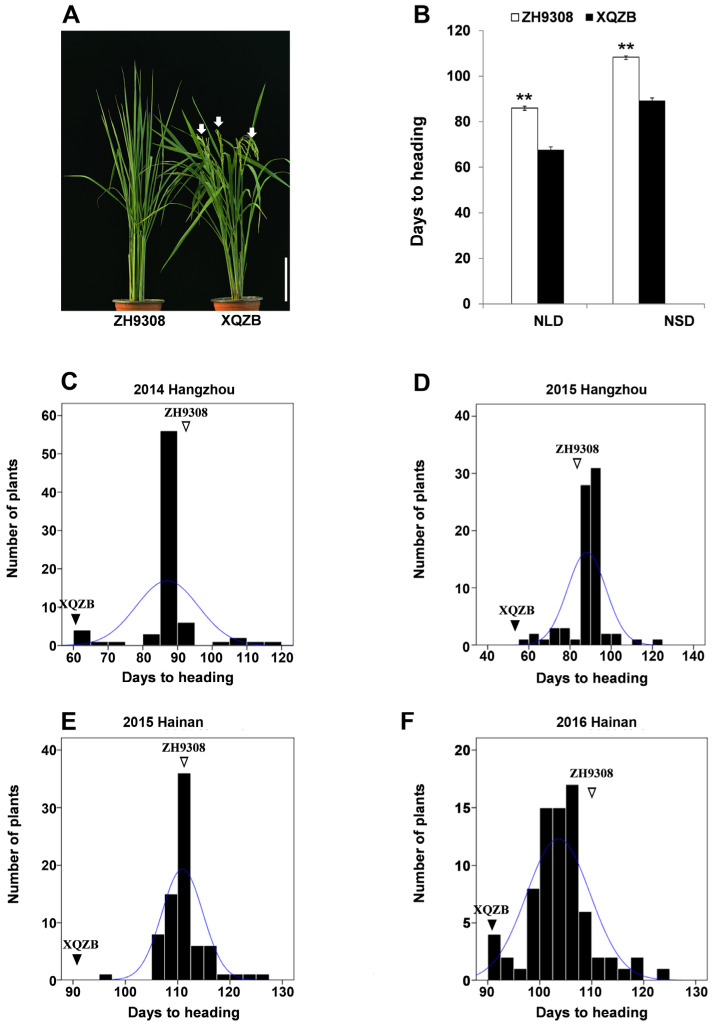
Comparison of ZH9308 and XQZB parents with ** indicating significant differences at *p* < 0.01 (**A**,**B**) and heading date distribution of the 76 chromosome segment substitution lines (CSSLs) based on the best linear unbiased prediction (BLUP) evaluated at the Hangzhou and Hainan growing conditions (**C**–**F**).

Seventy-six CSSLs and the two parents were evaluated for HD under natural SD (NSD) at Hainan in 2015–2016 and under natural LD (NLD) at Hangzhou in 2014–2015 for two seasons. The XQZB matured about 32 and 20 days earlier than the ZH9308 in the Hangzhou and Hainan growing conditions, respectively (Figure 1A,B; Table 1). The days to heading of 76 CSSLs exhibited 59 to 122 days in NLDs and from 93 to 124 days in NSDs (Figure 1C–F; Table 1). Overall, HD showed continuous variation in both growing conditions but skewed distribution (Table 1, Figure 1). The broad-sense heritability was computed from all four environments, Hainan, and Hangzhou, and were 0.83, 0.82, and 0.79, respectively (Table 1). PC1 and PC2 from the principal component analysis (PCA) accounted for 75.7% and 15.5%, respectively (Figure S2), with most CSSLs showing an average heading date clustered together at the origin. Highly-significant positive correlations were observed among the tested environments for heading date (Figure S2).

### 2.2. QTL Analysis of the CSSLs

The genetic linkage map of the CSSL population was constructed using 87 simple sequence repeats (SSRs) and 33 insertion/deletion (InDel) markers that followed the 1:1 Mendelian segregation pattern (Figure S3). A total of 120 markers were distributed across the whole genome with total coverage of 1311.02 cM using the Kosambi function of IciMapping software (Figure S3; Table S1). Inclusive composite interval mapping conducted on the BLUP HD data of the four environments using a LOD threshold value ≥ 2.5 identified 14 QTLs associated with heading date on Chr2 (*qHD2a*), Chr4 (*qHD4a* and *qHD4b*), Chr5 (*qHD5a*), Chr6 (*qHD6a* and *qHD6b*), Chr7 (*qHD7b* and *qHD7c*), Chr8 (*qHD8a*), Chr10 (*qHD10a* and *qHD10b*), Chr11 (*qHD11*), and Chr12 (*qHD12a* and *qHD12b*) (Table 2). The proportion of phenotypic variance (PVE) explained by each QTL ranged from 0.4% to 36.1%, and the additive effect from –15.4 to 18.2. Of the fourteen QTLs, only *qHD6a* and *qHD7b* were consistently detected in all four environments, from 10.1% to 36.1% (mean 23.14%) and from 8.1% to 32.8% (mean 20.5%), respectively. The remaining 12 QTLs were detected only in 1 or 2 environments (Table 2). We then focused on *qHD7b* for further research due to its consistent detection not only in all four environments in the present study, but also in two types of mapping populations in our previous study [23]. The favorable alleles of all QTL with a negative additive effect originated from the XQZB, while those with a positive allele originated from ZH9308. The XQZB allele at the *qHD7b* leads to early heading under both NSD and NLD conditions (Figure 1A,B; Table 2).

### 2.3. Photoperiodic Response of qHD7b

**Figure 2:**
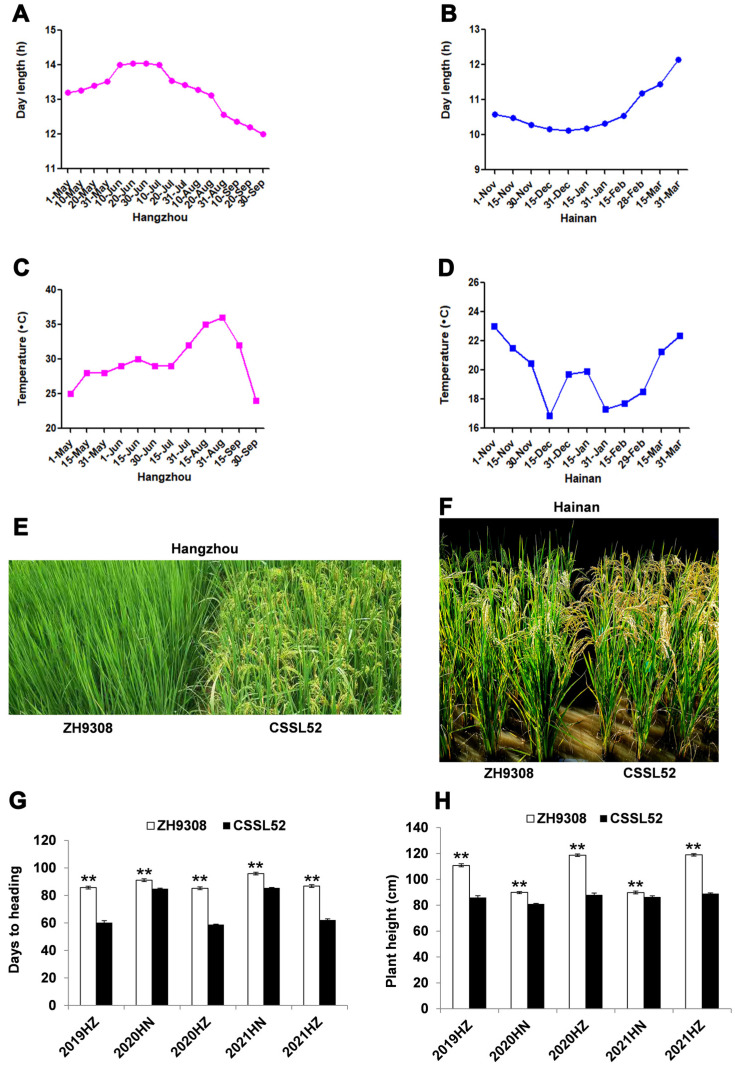
Comparison of heading date and plant height of ZH9308 and CSSL52 under five environmental conditions. (**A**–**D**) Daily photoperiod (**A**,**B**) and mean temperature (**C**,**D**) under Hangzhou and Hainan conditions in 2020 during the rice-growing season. (**E**,**F**) The phenotype of ZH9308 and CSSL52 under Hangzhou (**E**) and Hainan (**F**). The photo was taken at the CSSL52 heading stage in (**E**) and the CSSL52 maturation stage in (**F**). (**G**,**H**) Days to heading (**G**) and plant height (**H**) comparison between parents from 2019–2021 under NLD conditions in Hangzhou and NSD conditions in Hainan. The asterisks ** indicate significance between the parents at the *p* < 0.01, according to Student’s *t*-test.

The CSSL52 is one of the chromosome segment substitution lines, which contains two segments from XQZB in the ZH9308 background and harbors the early heading allele at the *qHD7b* region flanked by RM3859 and RM5875 markers (Figure S4). ZH9308 and CSSL52 were evaluated for five consecutive seasons under NLD and NSD conditions with different day lengths and temperatures (Figure 2A–D). There were significant HD differences (*p* < 0.01) between the parents in all the studied environments (Figure 2E–G). ZH9308 headed 24.6–26.4 days later than CSSL52 at Hangzhou NLD conditions (Figure 2E,G). At Hainan NSD conditions, ZH9308 headed 6.2–10.4 days later than CSSL52 (Figure 2F,G). Similarly, ZH9308 had a higher plant height than CSSL52 under all five environments (Figure 2H). Taken together, ZH9308 headed later than CSSL52 under both SD and LD conditions, and the phenotypic differences of days to heading were more significant under LD conditions. Due to longer days to heading, ZH9308 also exhibited a taller PH, longer panicle length, more numbers of internodes, longer internode length, a greater number of panicles per plant, and a more significant number of grains in the main panicle than CSSL52 (Figures S5 and S6). 

Heading date showed a highly-significant positive Pearson correlation with plant height (r = 0.83, *p* < 0.01), panicle length (r = 0.86, *p* < 0.01), and number of grains in the main panicle (r = 0.79, *p* < 0.01) (Table S2). Plant height showed high positive correlations with the panicle length (r = 0.83, *p* < 0.01) and number of grains in the main panicle (r = 0.80, *p* < 0.01). Similarly, there was also a significant positive correlation between panicle length and number of grains in the main panicle (r = 0.76, *p* < 0.01) (Table S2).

### 2.4. QTL Mapping in BC_5_F_2:3_ Population

The *qHD7b* was initially delimited to the 7.1 Mb region between the RM3859 and RM5875 markers (Table 1). We used a BC_5_F_2_ population to validate and fine map the *qHD7b* QTL, which was developed by crossing CSSL52 that has the early-heading allele with the ZH9308 parent and then backcrossing the progenies five times with the ZH9308 to develop a secondary F_2_ (BC_5_F_2_) population for fine mapping *qHD7b* (Figure S7). 

**Figure 3:**
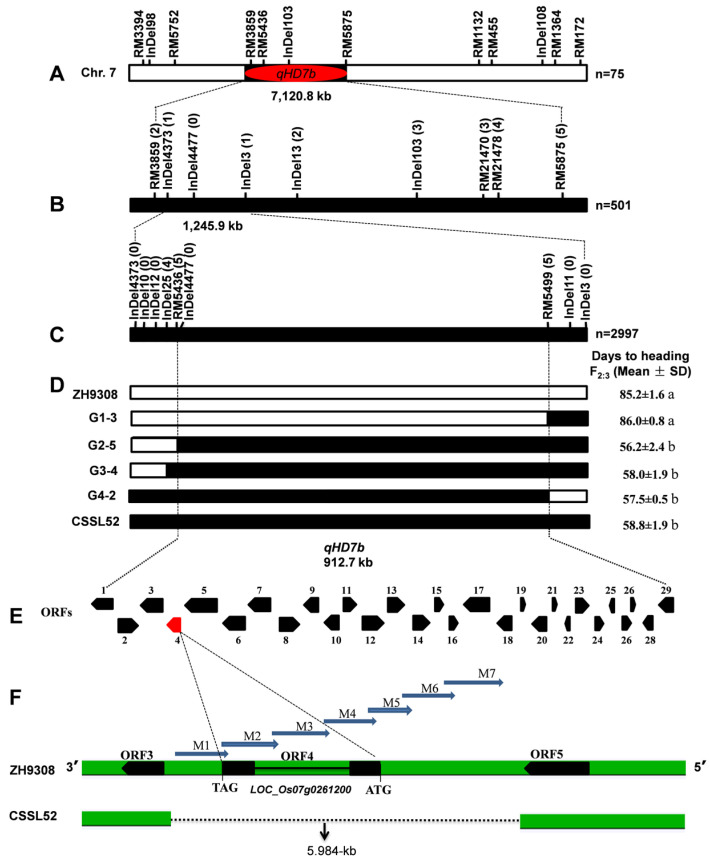
Coarse mapping and fine mapping of *qHD7b* on chromosome 7. (**A**) The position of *qHD7b* based on 76 CSSLs genotyped with 12 molecular markers on chromosome 7. (**B**) The position of *qHD7b* based on 501 BC_5_F_2_ plants genotyped with 7 markers located between RM3859 and RM5875 (the flanking markers identified using the CSSLs). (**C**) Fine mapping of *qHD7b* using BC_5_F_2:3_ plants genotyped with 7 markers that mapped between InDel4373 and InDel3 (the flanking markers identified using the 501 BC_5_F_2_ plants). (**D**) Genotypes and phenotypes of the two parents (ZH9308 and CSSL52) and 14 homozygous recombinant lines used for fine mapping of *qHD7b*. The ZH9308 and CSSL52 genotypic markers are represented by white and black bars, respectively. The 14 homozygous recombinants belong to four groups (G1 = 3 plants, G2 = 5 plants, G3 = 4 plants, and G4 = 2 plants). The superscripted letters (a and b) indicate statistically significant differences in the heading dates of recombinants relative to the parents. (**E**) Approximately 29 open reading frames (ORFs) were located between the two flanking markers identified during the fine mapping (RM5436 and RM5499), which are summarized in Table S4. (**F**) Sequence comparison between ZH9308 and CSSL52 with 5.984-kb deletion in CSSL52 at *Os07g0261200* using 7 markers (M1 to M7). The deleted region in CSSL52 is significant, and seven molecular markers (*Ghd7*-M1 to *Ghd7*-M7) linked with *qHD7b* are amplified in ZH9308.

Using a linkage map of 9 markers near the *qHD7b* QTL and heading data of a subset of 501 BC_5_F_2_ plants (Figure 3) evaluated for two consecutive seasons under Hangzhou NLD and two seasons under Hainan NSD conditions (Figure S8), we mapped the *qHD7b* QTL between the InDel4373 and InDel3 markers. The QTL was detected in all four environments (seasons) and was between 10.8% and 41.1% in each environment (mean = 26.0%), and had a LOD score ranging from 4.9 to 62.7 (mean = 33.8). Under Hangzhou conditions, however, the *qHD7b* had a very large effect (31.5–41.1%) and more significant LOD scores (59.8–62.7) than in the Hainan conditions (PVE = 10.8–21.2%, LOD = 4.9–7.5), which was about two-fold greater in the mean phenotypic effect and nearly ten-fold larger in the mean LOD score (Table S3). We then classified the 501 BC_5_F_2_ as late and early heading and calculated the mean difference in heading date. The average difference between the early and late segregating progenies was 9.6 and 25.5 days under NSD and NLD, respectively (Figure S8). A Chi-square analysis performed on the BC_5_F_2_ population fit the expected 3 (late):1 (early) ratio (386:115; χ^2^ = 0.52, *p* = 0.47), indicating that *qHD7b* behaves as a single dominant gene that is more functional under LD conditions than SD conditions.

### 2.5. Fine Mapping of qHD7b

To narrow down the confidence interval of the *qHD7b* identified using the 501 BC_5_F_2_ plants, we used a total of 2997 BC_5_F_2:3_ individuals genotyped with 7 markers that were polymorphic between the ZH9308 and CSSSL52, as well as the two flanking InDels (InDel4373 and InDel3) identified during the initial mapping. The genotype data revealed 14 homozygous recombinants (Figure 3D) that belong to 4 groups (G1 = 3 plants, G2 = 5 plants, G3 = 4 plants, and G4 = 2 plants). The recombinant plants in G1 had the same heading date (86.0 d) as the ZH9308 parent (85.2 d), while the remaining three recombinant groups had nearly the same heading date (56.2–58.0 d) as the CSSL52 parent (58.8 d). In the fine mapping, InDel4477, located at 9,075,693 bp, was the closest marker to *qHD7b*, while RM5436 and RM5499 were the flanking markers located at 9,075,636 bp and 9,988,139 bp on chromosome 7. In contrast to the G1 recombinants that had the ZH9308 parent genome between RM5436 and RM5499 at the *qHD7b* interval, the remaining three groups of recombinants (G2 to G4) all inherited the CSSL52 genome. The physical interval between RM5436 and RM5499 markers spans 912.7 kb (Figure 3D), which was confirmed using 20 BC_5_F_3:__4_ progeny from each recombinant group.

### 2.6. Candidate Gene Analysis of qHD7b and Validation Using CRISPR/Cas9

A candidate gene search using the physical position of the two flanking markers identified during the fine mapping (RM5436 and RM5499) in the Gramene database (https://www.gramene.org/ (accessed on 29 August 2022)) using the *Oryza sativa japonica* group reference genome identified 29 predicted genes, of which 18 had *Oryza indica* homologues that fell within the 912.7-kb region (7:9,075,636–9,988,139) of *qHD7b* (Figure 3E and Table S4). ORF4 (*Os07g0261200*) is physically located at 9,152,377 bp on chromosome 7, encodes the CCT motif family protein, and has been annotated as *Ghd7 (Os07g0261200)*. We then sequenced *Os07g0261200* in the two parents using seven markers (M1 to M7), which revealed a 5.984-kb deletion in the ORF4 region in the CSSL52 parent but not in ZH9308 (Figure 3F). The expression levels of *Os07g0261200* in CSSL52 were also nearly zero as compared with ZH9308, which also suggests that *Ghd7* is a probable putative candidate gene for *qHD7b* (Figure S9). 

**Figure 4:**
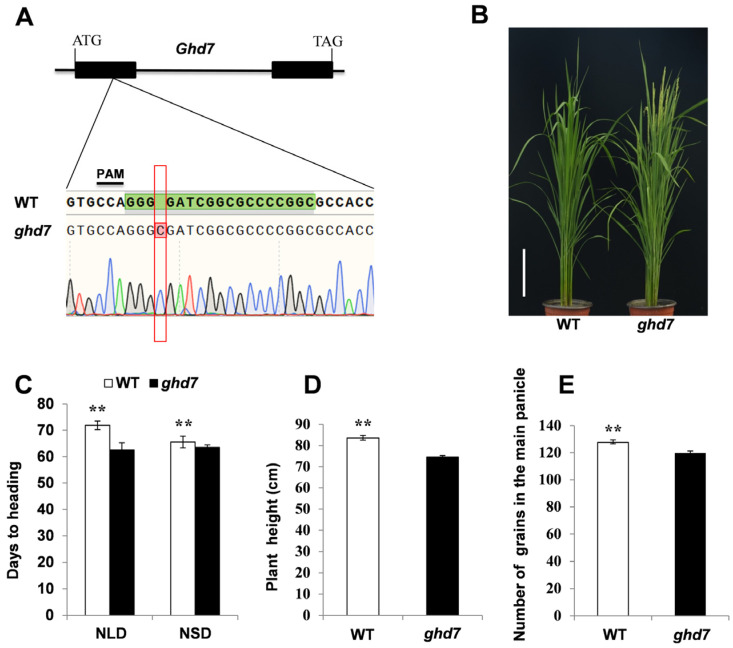
Heading date of wild-type (WT) and *ghd7* mutant in the Nipponbare genetic background. (**A**) Schematic of the *Ghd7* gene with the sgRNA:Cas9 targets (green) and corresponding protospacer-adjacent motif sequences (underlined). The insertion nucleotide is shown as a red letter. (**B**) The phenotype of WT and mutant *ghd7* at the heading stage under Hangzhou conditions. (**C**) Days to heading of WT and *ghd7* under natural long-day Hangzhou and natural short-day Hainan conditions. (**D**,**E**) Comparison of the WT and *ghd7* mutant for plant height (**D**) and the number of grains in the main panicle (**E**) under natural long-day conditions. The data are expressed as mean values ± SD. The asterisks ** indicate significance between WT and *ghd7* mutant at the *p* < 0.01, as determined by Student’s *t*-test.

To validate the mutant phenotype, we knocked out *Ghd7* in the Nipponbare genetic background utilizing the CRISPR/Cas9 system (Figure 4A). The HD of the wild type was 9.4 days later than the *ghd7* mutant under NLD conditions in Hangzhou (Figure 4B,C) and 2.0 days later under NSD conditions in Hainan (Figure 4C). The wild-type showed significant differences with the *ghd7* mutant for HD, plant height, and the number of grains per panicle (Figure 4D,E). 

## 3. Discussion

Heading date is one of the most important agronomic traits and varies widely in rice depending on the genetic differences among genotypes, environmental conditions, day length, temperature, and their interactions [23]. A clear understanding of the genetic drivers of heading dates is essential for cultivating rice in different geographical regions and seasons [24]. Numerous genetic mapping studies were conducted to identify genes and QTLs associated with heading using bi-parental populations, such as F_2_, backcross, doubled haploid lines, RILs, and CSSLs [25,26]. Hundreds of genomic regions related to HD have been reported in rice; however, few have been mapped and cloned [27]. *qHD7b* was identified using two types of populations that were 76 CSSLs and a BC_5_F_2_ population. Using 76 CSSL lines, the present study confirmed the *qHD7b* QTL that we previously reported between markers RM5436, located at 9,075,636 bp, and RM3670, located at 13,439,924 bp [22]. However, the physical interval of the QTL was about double in the CSSLs (7120.8 kb) as compared with the previous study (4364.3 kb), which is likely due to the small population size of the CSSLs. The use of 501 BC_5_F_2_ plants reduced the physical confidence interval of the QTL to 1245.9 kb, followed by 912.7 kb when it was fine-mapped using 2997 BC_5_F_2:3_ plants (Figure 3). RM5436 and RM5499 were the flanking markers of the *qHD7b* QTL after fine mapping, which were also previously reported as flanking markers for a major QTL (*qSSP7*), thereby controlling the number of spikelets per panicle [28], and another major QTL (*qSE7*), thereby influencing the stigma exertion rate [29] in rice. A candidate gene search conducted using the physical interval of the *qHD7b* (7:9,075,636–9,988,139) and the *Oryza sativa japonica* group reference genome in Gramene identified a total of 29 candidate genes. *Ghd7* (*Os07g0261200*) is one of the 29 candidate genes, which has been extensively studied for its effect on influencing heading date, grain yield, plant height, and other agronomic traits in rice [7,30]. However, other multiple genes should also be explored in the future.

One of the challenges in QTL mapping is identifying QTLs that are consistently detected across different environments, which was not the case for *qHD7b.* This QTL was consistently detected in all environments regardless of the type of mapping populations, but its effect and LOD scores tend to be very erratic. The LOD score for the *qHD7b* varied from 3.2 to 54.4 in the 76 CSSLs and from 4.9 to 62.7 in the 501 BC_5_F_2_ plants, while the phenotypic variance explained by the QTL varied from 8.1% to 32.8% in the CSSLs and from 10.8% to 41.1% in the BC_5_F_2_ plants (Table 2 and Table S3). A previous study showed that the QTL effect detected by the different mapping populations and environmental conditions is not necessarily the same [31]. In our case, the discrepancies may be due to differences in the population size and/or the type of mapping populations. CSSLs are powerful for QTL discovery studies due to the presence of multiple overlapping segments and several recombination events [32]. Still, the small size of the population in the current study may affect the QTL results. The BC_5_F_2_ population size, on the other hand, was ideal for QTL discovery studies in terms of population size but has limited recombination frequency, which makes it inferior in terms of mapping resolutions. The two phenotyping locations were also different in both temperature and day length, with Hangzhou’s showing a higher temperature and longer day length than Hainan (Figure 2A–D), which resulted in a clear difference in phenotype between the parents in Hangzhou’s than Hainan (Figure 2E–H) [33]. As a result, the proportion of phenotypic variance explained by *qHD7b* was very low in Hainan in both the CSSL and BC_5_F_2_ populations (Table 2 and Table S3). Liu et al. [23] also reported relatively low effects for *qHD1b* in Hainan than in Hangzhou. The differences in heading dates between the wild-type and the *ghd7* mutant were also smaller in Hainan than in the Hangzhou growing conditions, and recent studies also support this result [34]. These results suggest *qHD7b* as a major HD QTL function, mainly under LD conditions.

Our sequencing result revealed that at the *Ghd7* locus, the *qHD7b*^XQZB^ allele belongs to *Ghd7*-0, which is non-functional, and the *qHD7b*^ZH9308^ allele belongs to *Ghd7*-4, which is functional together with *Ghd7*-1, *Ghd7*-2, and *Ghd7*-3 [35]. The non-functional Minghui 63 allele of *Ghd7* showed non-significant phenotype differences under SD conditions [7]. However, under SD conditions, the *qHD7b*^XQZB^ allele flowered earlier than the *qHD7b*^ZH9308^ allele (Figure 2G). These phenotypic differences may be caused by background differences, indicating *qHD7b* also functions in SD conditions, but the effect is smaller than that in LD conditions. The two flanking molecular markers for *qHD7b* (RM5436 and RM5499) and the seven molecular markers that mapped within the QTL confidence interval (*Ghd7*-M1 located at 9,150,263 to *Ghd7*-M7 located at 9,155,572 bp) can contribute towards the effort in the breeding of rice varieties using marker-assisted selection (MAS). For example, *qHD7b*^ZH9308^ could be useful in breeding late-maturing cultivars. When the rice cultivars with *Ghd7*-0a, *Ghd7*-0, and *Ghd7*-2 alleles originating from northern China were introduced to southern China, their heading date would be significantly earlier. The *qHD7b*^ZH9308^allele could be introduced into these cultivars to prolong their heading date to make them have delayed heading and increase their yield. On the contrary, *qHD7b*^XQZB^ could be useful in breeding early-maturing cultivars. When the rice cultivars with *Ghd7*-1, *Ghd7*-3, and *Ghd7*-4 alleles originating from the tropical and subtropical regions were introduced to northern China, their grains couldn’t reach maturity due to later heading. The *qHD7b*^XQZB^ allele could be introduced into these cultivars to make early heading to mature and harvest in time.

## 4. Materials and Methods 

### 4.1. Population Development and Phenotyping 

The present study was conducted using three populations. One of the populations was developed by crossing 134 RILs with Zhonghui9308 (ZH9308), which was then backcrossed three times, and selfed six times to form BC_4_F_6_ generation. The RILs were initially developed from a cross between an early-heading XieqingzaoB (XQZB) donor parent and a late-heading ZH9308 recipient parent and parental lines of Xieyou9308 (an *indica*-*japonica* subspecies super hybrid rice with 87.5% *indica* and 12.5% *japonica* genome) [22]. Seventy-six of the 134 BC_4_F_6_ lines were selected to represent CSSLs for QTL mapping, and the genotypes of the 76 CSSLs were also investigated [36]. The second population was developed by crossing one of the CSSLs (CSSL52) that exhibited early heading with the ZH9308 parent and then backcrossing the progenies four times with the ZH9308 to develop a secondary BC_5_F_2_ population for validation and a BC_5_F_2:3_ population for the fine-mapping of the *qHD7b* QTL (Figure S1). 

A set of 76 CSSLs along with parental lines, ZH9308 and XQZB, were evaluated for heading date through a Randomized Complete Block (RCB) design consisting of 3 replications, with 4 rows × 8 plants for each line at Hangzhou and Hainan for 4 seasons. Each plot consisted of 4 rows of 1.32 square meters with 16.5 cm × 26.5 cm spacing between plants and rows. The BC_5_F_2_ population, along with ZH9308 and CSSL52 parents, was evaluated for two years under NLD (day length >14 h) conditions in Hangzhou, Zhejiang Province (120.0° E, 30.15° N), and NSD (day length <12 h) conditions in Lingshui, Hainan Province (110.0° E, 18.5° N). HD was recorded as the number of days from the sowing date to the emergence of the first heading. Plant height (PH) was measured from the ground level to the tip of the panicle at full physiological maturity. Internode length was recorded as the length between two nodes. Number of panicles per plant was recorded as the number of all panicles per plant. Panicle length (PL) was recorded from the neck to the apex of the panicle. The number of grains in the main panicle was recorded as the filled grain numbers of the main panicle. Paddy field management followed conventional practices [37].

### 4.2. Test of Day Length Response in Growth Chambers

To investigate the photoperiodic response, ZH9308 and CSSL52 plants were grown under CLD (14 h light, 30 °C/10 h darkness, 25 °C) and CSD (10 h light, 30 °C/14 h darkness, 25 °C). The humidity was 75%, and the light intensity was 300 μmol m^−2^ s^−1^ [38]. 

### 4.3. Molecular Markers Development and DNA Extraction 

Polymorphisms between ZH9308 and CSSL52 were screened using Insertion/Deletion (InDel) and Simple Sequence Repeat (SSR) markers [39]. To determine the candidate genes of *qHD7b* QTL, the sequence between RM3859 and RM5875 was downloaded from the EnsemblePlants (http://plants.ensembl.org/index.html (accessed on 29 August 2022)) and Gramene website (https://www.gramene.org/ (accessed on 29 August 2022)) using the *Oryza sativa japonica* group reference genome by a blast search of the primer sequences. New InDel markers in the QTL region were designed based on differences in genomic sequence between Indica and Japonica using the Primer Premier 5.0 software (PREMIER Biosoft International, San Francisco, CA, USA). The markers are listed in Tables S5 and S6. The markers that amplified the mutated region between parents are listed in Table S7. 

Genomic DNA was extracted from the fresh leaves of parents and the secondary F_2_ (BC_5_F_2_) population using the cetyltrimethylammonium bromide (CTAB) method described by Luo et al. [40]. A polymerase chain reaction (PCR) was performed in a 12 µL volume that consisted of 5 µL of master mix, 3 µL of ddH_2_O, 2 µL of 10 pmol µL^−1^ primers (1 µL forward primer and reverse primers), and 2 µL of template genomic DNA (200 ng). The PCR amplification protocol consisted of a pre-denaturation step (95 °C for 3 min), followed by 32 cycles (denaturation at 95 °C for 15 s, annealing at 55 °C for 15 s, and extension at 72 °C for 5 s), and final extension (72 °C for 10 min). The PCR products were separated using gel electrophoresis on 8% non-denaturing polyacrylamide gel and visualized with silver nitrate staining using a formaldehyde solution [41]. 

### 4.4. RNA Extraction and qRT-PCR

The total RNA was extracted from leaves of ZH9308 and CSSL52 using an RNAprep Pure Plant kit (Tiangen Biotech Co., Ltd., Beijing, China). A total of 50 µL of complementary DNA (cDNA) was synthesized using 5 µg of RNA with ReverTra Ace^®^ qPCR RT Master Mix with gDNA Remover (Toyobo Co., Ltd., Osaka, Japan). Real-time quantitative RT-PCR (RT-qPCR, 20 µL reaction volume) was performed using 0.5 µL of cDNA, 0.2 µM of each gene-specific primer, and TB Green Premix ExTaq II (Takara Bio, Inc., Kusatsu, Shiga, Japan) in a LightCycler^®^480 II (Roche). The rice *Ubq* gene (*Os03g0234350*) was exploited as the endogenous control. The primers for qRT-PCR were designed using GeneScript (https://www.genscript.com/tools/real-time-pcr-taqman-primer-design-tool (accessed on 29 August 2022)) and are displayed in Table S7.

### 4.5. Generation of ghd7 Mutant Using CRISPR/Cas9 System

The CRISPR/Cas9 system was used to knock out *Ghd7* according to the methods as described previously [42]. The 18-bp sgRNA:Cas9 target sequence of *Ghd7* was introduced into the pCas9-sgRNA vector at the *AarI* site. The final vector was transformed into Nipponbare using *Agrobacterium*-mediated transformation [43]. The primers used are displayed in Table S7.

### 4.6. Statistical Analysis 

The experimental design in each environment was a randomized complete block design with three replications per environment/location. The best linear unbiased prediction (BLUP) values were obtained through META-R v6.03 [44], using the linear model:

Y_ik_ = µ + Rep_i_ + Gen_k_ + ε_ik_ (within the environment)

Y_ijk_ = µ + Rep_i_ (Env_j_) + Env_j_ × Gen_k_ + Gen_k_ + Env_j_ + ε_ijk_ (across environments)

where Y_ik_ is the trait of interest, µ is the mean effect, Rep_i_ is the effect of the _i_th replicate, Gen_k_ is the effect of the _k_th genotype, ε_ik_ is the error associated with the _i_th replication, and the _k_th genotype, which is assumed to be normally and independently distributed. For across environments, Y_ijk_ is the trait response, Env_j_ is the _j_th environment, Rep_i_ (Env_j_) is the effect of _i_th replication in the _j_th environment, and Env_j_ × Gen_k_ is the environment and genotype interaction. The resulting analysis produced the adjusted trait phenotypic values in the form of BLUP within and across environments. The BLUP model considers genotypes as random effects. Broad sense heritability (H^2^) and repeatability (H) were calculated according to Alemu et al. [45] using META-R software.

$$H=\frac{\sigma^{2}g}{\sigma^{2}g+\sigma^{2}e/\mathrm{reps}}\;(\mathrm{within}\;\mathrm{the}\;\mathrm{environment})$$

$$H=\frac{\sigma^{2}g}{\sigma^{2}g+\sigma^{2}\mathrm{ge}/\mathrm{env}+\sigma^{2}e/(\mathrm{reps}\times \mathrm{env})}\;(\mathrm{across}\;\mathrm{environment})$$

where σ^2^g and σ^2^e are the genotypic and error variance, σ^2^ge is the genotype by environment interaction variance, rep is the number of replicates, and env is the number of environments.

QTL analysis was performed using the inclusive composite interval mapping (ICIM) function implemented in QTL IciMapping software [46]. A LOD threshold value ≥2.5 indicates the presence of QTL (selected by 1000 permutation tests to obtain a 0.05 genome-wide probability level of Type I error, with a search step of 1 cm). QTLs were named by placing a “q” at the beginning of the trait “HD”, followed by the chromosome number. For more than one QTL on the same chromosome, a second identifier was placed after the chromosome number reported previously [47]. 

## 5. Conclusions

A total of fourteen significant (LOD ≥ 2.5) HD QTLs (*qHD2a*, *qHD4a*, *qHD4b*, *qHD5a*, *qHD6a*, *qHD6b*, *qHD7b*, *qHD7c*, *qHD8a*, *qHD10a*, *qHD10b*, *qHD11a*, *qHD12a*, and *qHD12b*) were detected in the 76 CSSL populations concerning the position and introgression segments under four different environments. We focused on *qHD7b* for further research due to its stability. A secondary F_2_ (BC_5_F_2_) population was developed by backcrossing CSSL52 with recurrent parent ZH9308, and *qHD7b* was narrowed down to the 912.7-kb region, flanked by markers RM5436 and RM5499 using 2995 individuals from the secondary F_2:3_ (BC_5_F_2:3_) population. The CSSL52 allele at the *qHD7b* locus negatively regulates HD under SD and LD conditions. Sequencing and expression analysis demonstrated that *Os07g0261200* encodes *Ghd7*, a suitable candidate gene for *qHD7b*. The *ghd7* mutant generated through CRISPR/Cas9 promoted the heading date and validated *Ghd7* as a putative candidate gene for HD. Further study on *qHD7b* will contribute to MAS and the developing late-maturing varieties that can be used in diverse geographical regions.

## Figures and Tables

**Table 1:** Summary of heading dates of parents and 76 chromosome segment substitution lines (CSSLs) evaluated under two natural short-day conditions at Hainan and long-day conditions at Hangzhou.

	Parents ^b^		76 CSSLs ^a^						
Year/Location	ZH9308	XQZB	Min	Max	Mean	SD	Kurtosis	Skewness	H
2014 Hangzhou	91.83 ± 1.11 **	60.17 ± 1.10	60.40	119.22	87.12	8.89	5.21	−0.23	0.79
2015 Hangzhou	88.33 ± 0.50 **	56.67 ± 0.54	57.67	124.28	88.21	9.42	4.45	−0.33	
2015 Hainan	110.99 ± 1.52 **	91.00 ± 1.31	96.57	124.94	110.87	3.94	4.33	0.63	0.82
2016 Hainan	109.67 ± 1.24 **	91.44 ± 1.67	90.00	122.80	103.59	6.10	1.54	0.36	
**Combined**									
	**Trait**	**Min**	**Max**	**Mean**	**σ^2^G**	**σ^2^GxE**	**σ^2^E**	**CV**	**H^2^**
	HD	80.77	119.78	97.45	34.86	24.91	8.98	3.08	0.83

^a^ Values for CSSLs are minimum (Min), maximum (Max), mean, standard deviation (SD), and repeatability (H^2^). ^b^ Data of parents are presented as mean ± SD with ** indicating significant differences between ZH9308 and XQZB at the *p* < 0.01.

**Table 2:** Chromosomal locations of putative HD QTLs under four different environments using 76 CSSL populations.

QTLs	Year/Location ^a^	Chr.	Region (cM)	Flanking Markers	LOD ^b^	PVE (%) ^c^	Add ^d^
*qHD2a*	2015HZ	2	19.77	RM6424-InD31	2.80	2.54	2.37
*qHD4a*	2014HZ	4	6.58	InD62-RM1205	38.89	9.87	13.13
	2015HZ	4	10.54	RM1205-RM5979	6.46	4.50	1.75
*qHD4b*	2015HZ	4	21.08	RM3839-RM241	11.33	9.14	8.79
*qHD5a*	2014HZ	5	25.20	RM3638-RM6841	2.59	0.45	−0.47
	2016HN	5	14.52	InD79-RM3638	2.91	7.78	−3.91
*qHD6a*	2014HZ	6	6.58	RM5754-RM5963	49.67	19.03	18.21
	2015HN	6	5.27	RM510-RM5754	11.12	36.14	6.78
	2015HZ	6	6.58	RM5754-RM5963	13.14	10.13	9.90
	2016HN	6	5.27	RM510-RM5754	8.83	25.01	8.67
*qHD6b*	2014HZ	6	11.85	RM20069-InD94	3.77	0.33	0.36
*qHD7b*	2014HZ	7	27.69	RM3859-RM5875	54.42	26.54	−15.44
	2015HN	7	27.69	RM3859-RM5875	3.24	8.10	−2.61
	2015HZ	7	27.69	RM3859-RM5875	26.56	32.84	−12.90
	2016HN	7	27.69	RM3859-RM5875	7.55	20.33	−6.76
*qHD7c*	2015HZ	7	21.13	RM1132-RM455	2.56	2.79	0.88
*qHD8a*	2014HZ	8	7.91	RM5556-RM22529	46.40	16.12	−12.03
	2015HZ	8	7.91	RM5556-RM22529	15.91	13.30	−8.22
*qHD10a*	2015HZ	10	2.63	InD133-InD135	12.27	9.14	−13.28
	2015HN	10	2.63	InD133-InD135	6.67	18.74	−7.37
*qHD10b*	2016HN	10	6.58	RM6142-RM5620	3.60	8.89	−5.96
*qHD11a*	2014HZ	11	10.54	RM7463-RM26652	18.14	1.94	−7.76
	2015HZ	11	17.16	RM26652-InD151	2.51	3.10	−2.72
*qHD12a*	2014HZ	12	2.63	InD156-RM7003	15.42	2.21	−6.10
*qHD12b*	2014HZ	12	23.82	InD165-RM1300	2.85	0.43	−0.44

^a^ Year/Location refers to the year of the experiment (2014, 2015, and 2016) followed by the location (Hangzhou—HZ/Hainan—HN). ^b^ Logarithm of odd, ^c^ the proportion of the phenotypic variance explained by the QTL effect, ^d^ the sign of the additive effects shows the parental origin of the favorable alleles (negative = XQZB and positive = ZH9308).

## Data Availability

Not applicable.

## Supplementary Materials

The following supporting information can be downloaded at: https://www.mdpi.com/article/10.3390/plants11172288/s1, Figure S1: Breeding scheme for QTL identification and fine mapping, Figure S2: GGE Biplot of days to heading from 76 CSSLs tested in four environments, Figure S3: Linkage map of 76 CSSLs derived from ZH9308 × XQZB using 120 polymorphic markers, Figure S4: A schematic representation of ZH9308, XQZB, and CSSL52 plants to show differences on chromosome 7 near *qHD7b*, Figure S5: Agronomic trait phenotypes of ZH9308 and CSSL52, Figure S6: Measurement of agronomic traits of ZH9308 and CSSL52, Figure S7: Phenotypes and genotypes of ZH9308, CSSL52, and secondary F_2_ (BC_5_F_2_) population, Figure S8: Frequency distribution of heading date in the secondary F_2_ (BC_5_F_2_) population under Hainan and Hangzhou conditions, Figure S9: The expression levels of *Os07g0261200* in ZH9308 and CSSL52, Table S1: Chromosome-wise SNP markers and genetic map length of rice CSSL population, Table S2: Pearson correlation coefficients among heading date and yield-related traits, Table S3: Evaluation of heading date QTL *qHD7b* under Hainan and Hangzhou conditions, Table S4: Candidate genes within 912.7-kb physical regions of *qHD7b* on chromosome 7, Table S5: Polymorphic DNA markers used in heading-date QTL analysis in 76 CSSLs and the secondary F_2_ (BC_5_F_2_) population, Table S6: DNA markers used in QTL analysis and fine mapping of *qHD7b*, Table S7: Markers used for sequencing, qRT-PCR, and CRISPR.
