# Supplementary material for: Mapping and Validation of qHD7b: Major Heading-Date QTL Functions Mainly under Long-Day Conditions
Source: Plants (Basel). 2022 Sep 1;11(17):2288. doi: 10.3390/plants11172288 (PMC9459803; doi:10.3390/plants11172288)
Supplement: Supplementary file 1 [file plants-11-02288-s001.zip › plants-1814425-supplementary.pdf]

plants

MDPI

Article

Mapping and Validation of *qHD7b:* Major Heading-Date QTL Functions Mainly Under Long-Day Conditions

Amir Sohail [1], Liaqat Shah [1,2], Ling Liu [1], Anowerul Islam [1,3], Zhengfu Yang [1,4], Qinqin Yang [1], Galal Bakr Anis [1,5], Peng Xu [1], Riaz Muhammad Khan [1,6], Jiaxin Li [1], Xihong Shen [1], Shihua Cheng [1], Liyong Cao [1,7], Yingxin Zhang [1,*] and Weixun Wu [1,*]

Supplementary materials

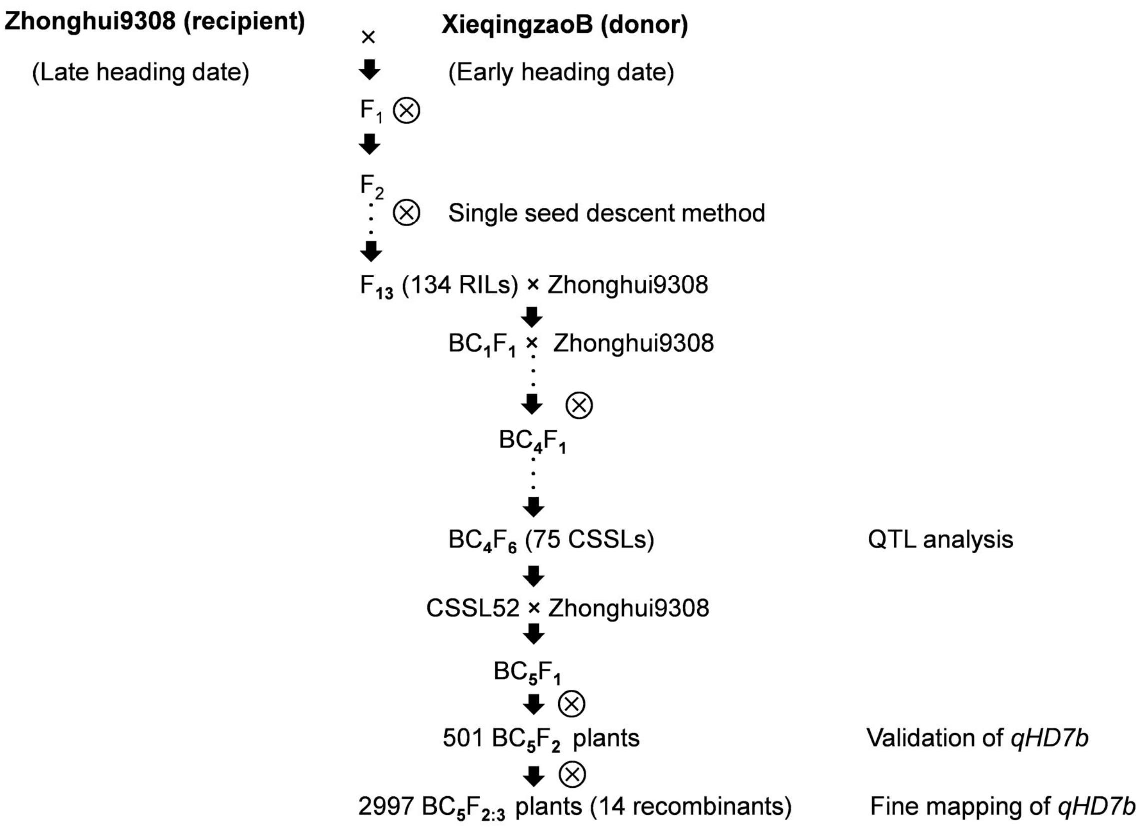

Figure S1. Breeding scheme for QTL identification and fine mapping.

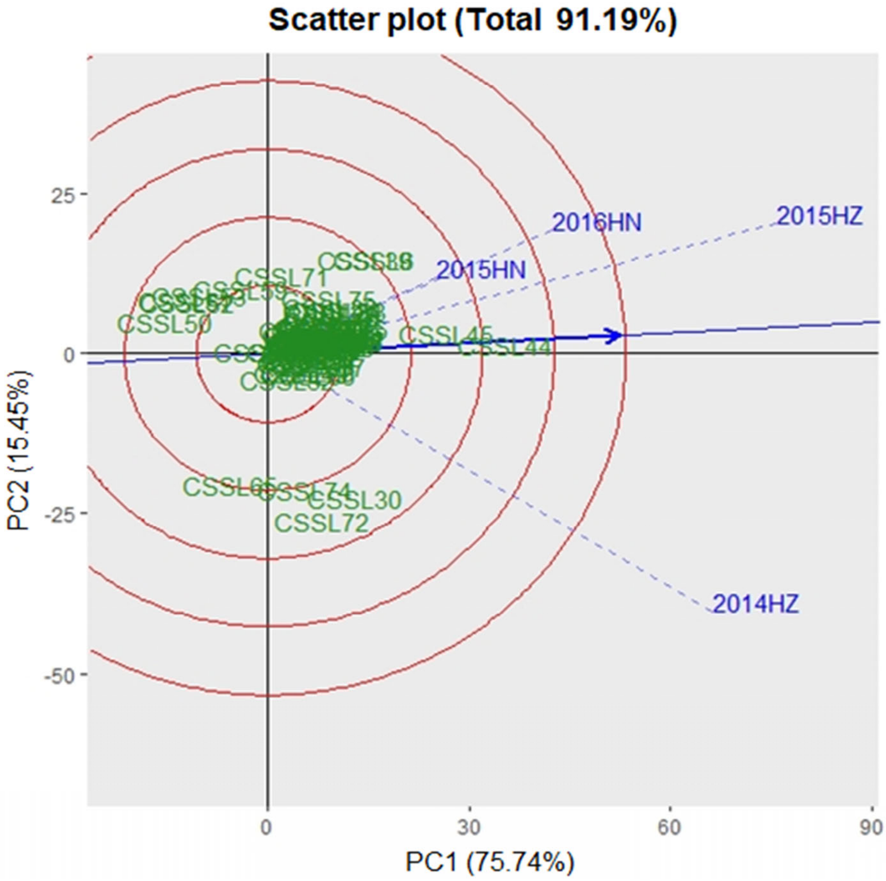

Figure S2. GGE Biplot of days to heading from 76 CSSLs tested in four environments.

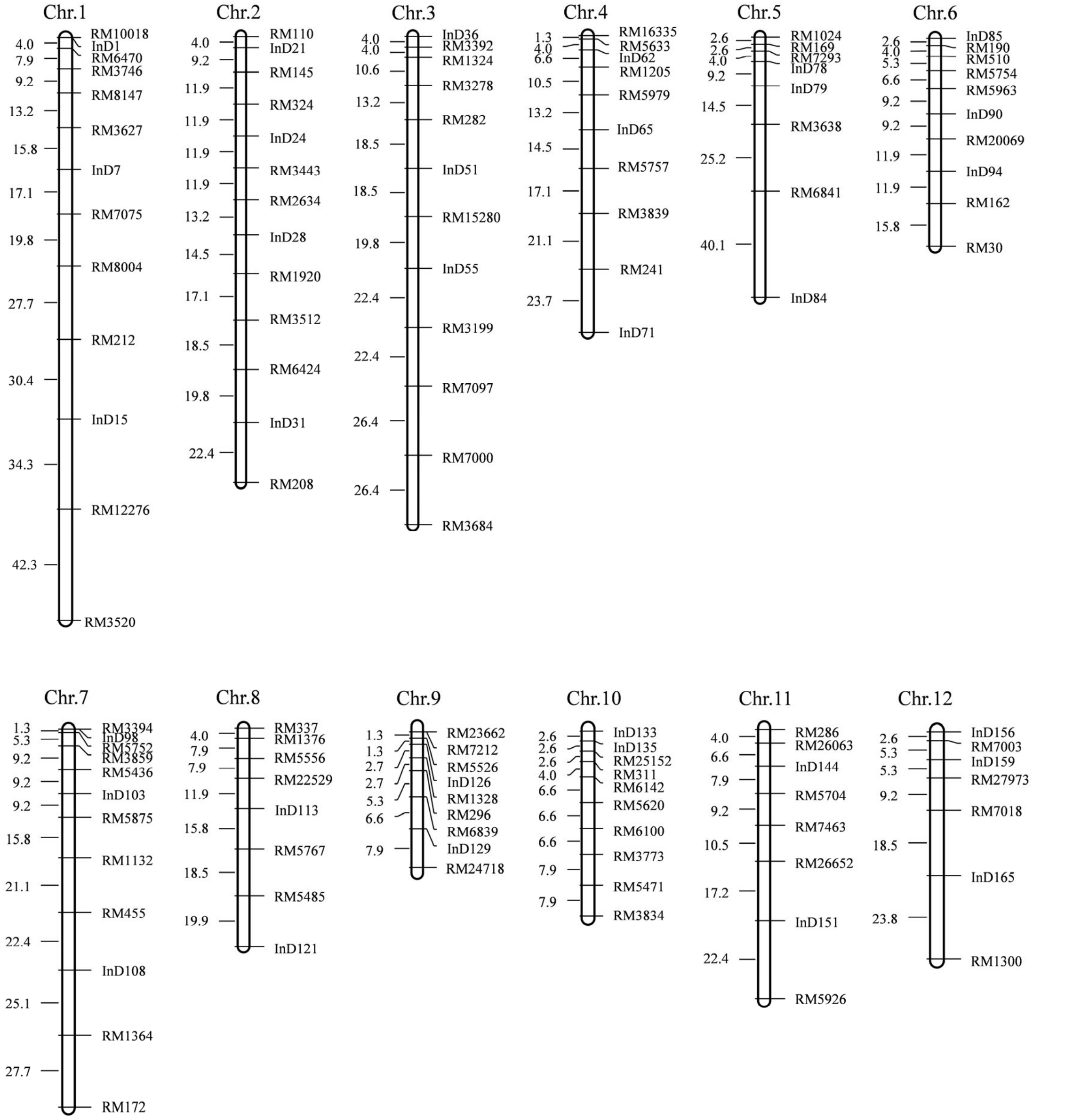

Figure S3. Linkage map of 76 CSSLs derived from ZH9308 × XQZB using 120 polymorphic markers. Markers' positions were calculated in centimorgan (cM) and positioned from the top of each linkage group.

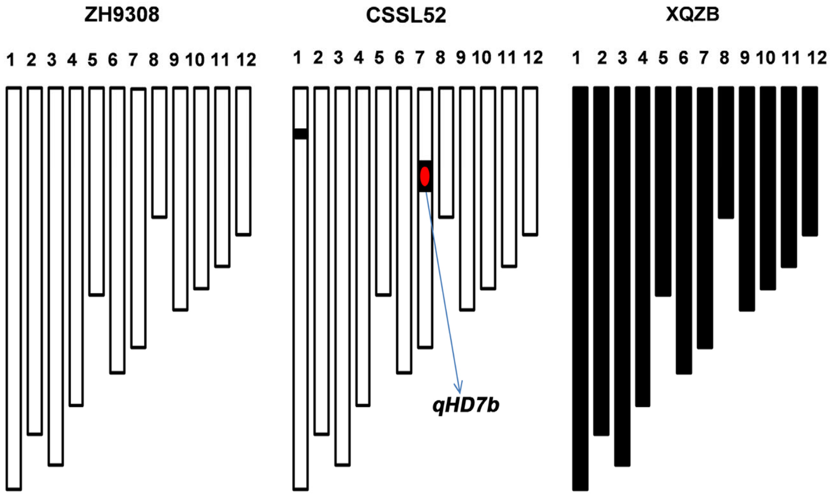

Figure S4. A schematic representation of ZH9308, XQZB, and CSSL52 plants to show differences on chromosome 7 near *qHD7b*. The 12 chromosomes are presented as vertical bars and numbered at the top. In the CSSL52 chromosomal map, the black region indicates the XQZB introgressed segment in the ZH9308 background, and the red circle indicates the *qHD7b* locus.

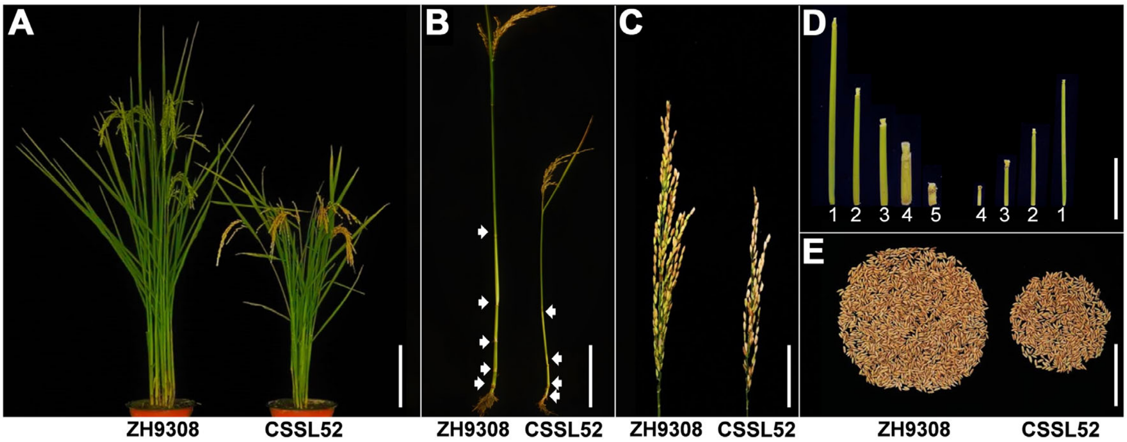

Figure S5. Agronomic trait phenotypes of ZH9308 and CSSL52. (**A**) Phenotypes of ZH9308 and CSSL52 at the CSSL52 maturation stage. (**B**) Main culms of ZH9308 and CSSL52. (**C**) Main panicle of ZH9308 and CSSL52. (**D**) Internode length of ZH9308 and CSSL52. (**E**) Grain yield per plant of ZH9308 and CSSL52. Bar = 20 cm.

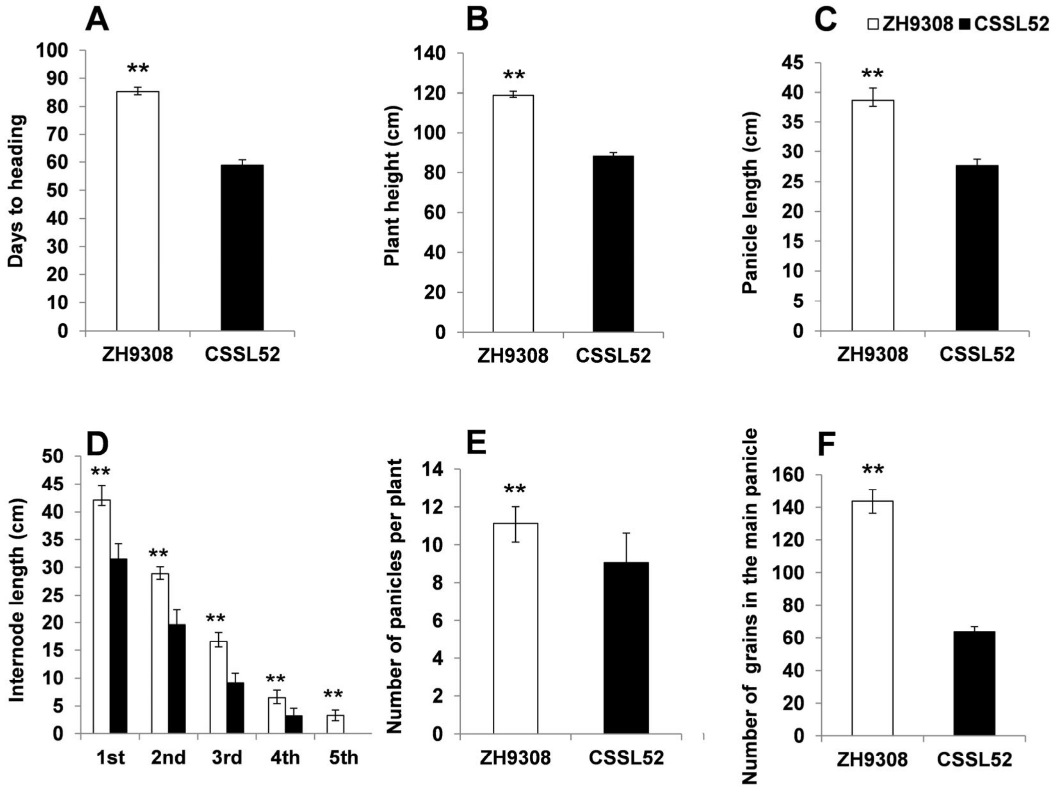

Figure S6. Measurement of agronomic traits of ZH9308 and CSSL52. (**A**) days to heading, (**B**) plant height, (**C**) panicle length, (**D**) internode length, (**E**) number of panicles per plant, and (**F**) number of grains in the main panicle. Sixteen plants (n = 16) were used for measuring agronomic traits. The asterisks ** indicate significance at the $P < 0.01$, according to Student's *t*-test.

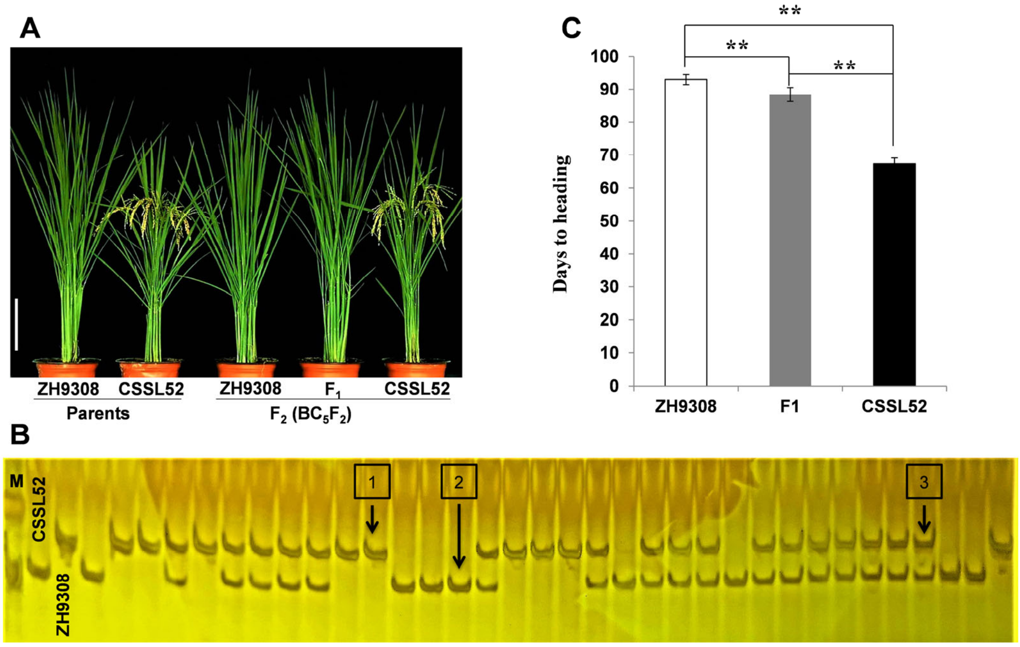

Figure S7. Phenotypes and genotypes of ZH9308, CSSL52, and secondary F_2 (BC_5F_2) population. (**A**) The phenotypes of parental lines ZH9308, CSSL52, and secondary F_2 (BC_5F_2) population. (**B**) Genotypes of ZH9308, CSSL52, and secondary F_2 (BC_5F_2) population used the InDel11 marker. Number 1 indicates the genotypes of the homozygous ZH9308 allele, number 2 indicates the homozygous XQZB allele, and number 3 indicates the heterozygous allele. (**C**) Days to heading of ZH9308, F_1, and CSSL52 under NLD conditions. The asterisks ** indicate significant differences at the $P < 0.01$, according to Student's *t*-test.

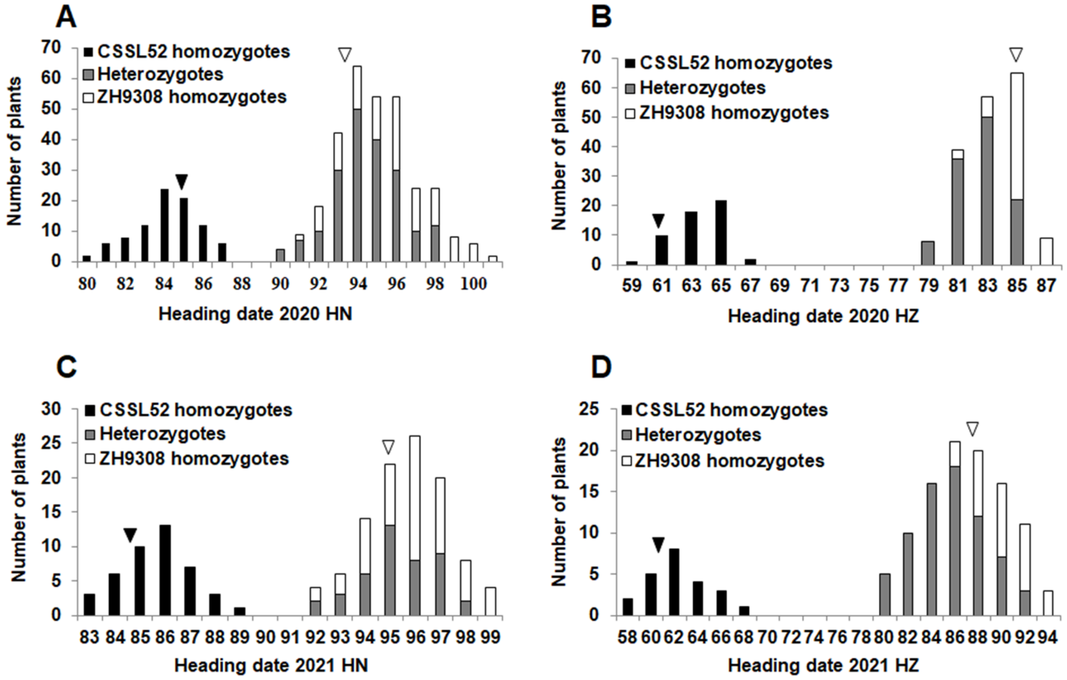

Figure S8. Frequency distribution of heading date in the secondary F_2 (BC_5F_2) population under Hainan and Hangzhou conditions. The black and white arrows indicate the average HD of CSSL52 and ZH9308.

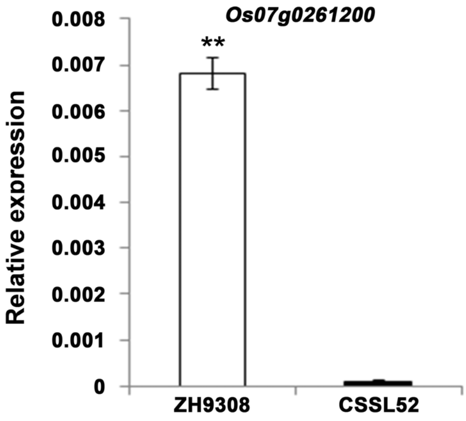

Figure S9. The expression levels of *Os07g0261200* in ZH9308 and CSSL52. Data presented as means ± SD were obtained from three technical and two biological replicates. The asterisks ** indicate significant differences at the $P < 0.01$.

Table S1. Chromosome-wise SNP markers and genetic map length of rice CSSL population.

Chr.	Markers			Map distance (cM)
	InDel	SSR	Total	
Chr. 1	3	10	13	221.7
Chr. 2	4	9	13	166.1
Chr. 3	3	9	12	186.2
Chr. 4	3	7	10	112.0
Chr. 5	3	5	8	98.3
Chr. 6	3	7	10	76.4
Chr. 7	3	9	12	146.5
Chr. 8	2	6	8	85.9
Chr. 9	2	7	9	27.8
Chr. 10	2	8	10	47.4
Chr. 11	2	6	8	77.8
Chr. 12	3	4	7	64.8
Total	33	87	120	1310.7

Table S2. Pearson correlation coefficients among heading date and yield-related traits.

Traits	Days to heading	Plant height	Panicle length
Plant height	0.83**		
Panicle length	0.86**	0.83**	
Number of grains in the main panicle	0.79**	0.80**	0.76**

The asterisks ** indicate statistically significant differences at the $P < 0.01$.

Table S3. Evaluation of heading date QTL *qHD7b* under Hainan and Hangzhou conditions.

Year/Location	QTL name	Marker interval	Region (cM)	LOD[a]	A[b]	PVE (%)[c]
2020 Hainan	*qHD7b*	InDel4373-InDel3	17.0	4.85	–2.79	10.75
2021 Hainan	*qHD7b*	InDel4373-InDel3	17.0	7.49	–1.45	21.18
2020 Hangzhou	*qHD7b*	InDel4373-InDel3	17.0	62.72	–9.33	41.07
2021 Hangzhou	*qHD7b*	InDel4373-InDel3	17.0	59.75	–7.63	31.50

[a] Logarithm of odd, [b] additive effect, [c] the proportion of phenotypic variance explained by the QTL effect.

Table S4. Candidate genes within 912.7-kb physical regions of *qHD7b* on chromosome 7.

ORFs	Gene ID (*Oryza sativa*)	Location	Gene ID (*Oryza indica* homologues)	Location	Putative function
ORF1	Os07g0260000	9075904-9077790	BGIOSGA024504	9088279-9093394	CRR4, putative, expressed
ORF2	Os07g0260300	9099097-9102892	BGIOSGA025480	9123440-9126788	Peroxiredoxin, putative, expressed
ORF3	Os07g0260400	9105495-9112919	BGIOSGA024503	9130176-9136660	Phospholipase D, putative, expressed
ORF4	Os07g0261200	9152377-9155030	BGIOSGA024502	9172628-9175046	CCT motif family protein, expressed
ORF5	Os07g0261732	9207189-9207535	No homologues		Similar to 10A19I.4.
ORF6	Os07g0262200	9221537-9227207	BGIOSGA024501	9250364-9255694	Mitochondrial prohibitin complex protein 2, putative, expressed
ORF7	Os07g0262300	9239129-9242275	No homologues		Conserved hypothetical protein
ORF8	Os07g0262600	9252350-9254375	No homologues		Legume lectins beta domain-containing protein, expressed
ORF9	Os07g0262700	9252390-9254389	No homologues		Hypothetical protein
ORF10	Os07g0262800	9260544-9262830	BGIOSGA024500	9289301-9290605	Legume lectins beta domain-containing protein, expressed
ORF11	Os07g0263400	9298577-9300441	No homologues		Erythronate-4-phosphate dehydrogenase domain-containing protein, expressed
ORF12	Os07g0264000	9344631-9349461	No homologues		Expressed protein
ORF13	Os07g0264100	9353167-9357704	BGIOSGA025486	9359119-9363508	Erythronate-4-phosphate dehydrogenase domain-containing protein, expressed
ORF14	Os07g0264800	9395365-9397525	BGIOSGA025487	9399246-9401156	Acetyltransferase, GNAT family, putative, expressed
ORF15	Os07g0264900	9399351-9403697	BGIOSGA025488	9403454-9407373	FAD binding protein, putative, expressed
ORF16	Os07g0265100	9405839-9407139	BGIOSGA025489	9409762-9410903	Expressed protein
ORF17	Os07g0265600	9460566-9467850	BGIOSGA024499	9448013-9458964	Piwi domain-containing protein, putative, expressed
ORF18	Os07g0267200	9538087-9541917	No homologues		Expressed protein
ORF19	Os07g0267300	9564958-9565455	No homologues		Expressed protein
ORF20	Os07g0267400	9568413-9573289	BGIOSGA037560	17925936-17929828	Peptidase C48, SUMO/Sentrin/Ubl1 domain-containing protein
ORF21	Os07g0268800	9738390-9740798	BGIOSGA033015	14171194-14173446	Expressed protein
ORF22	Os07g0269400	9820628-9821346	No homologues		Expressed protein
ORF23	Os07g0269450	9824468-9831477	No homologues		Hypothetical protein
ORF24	Os07g0269800	9858212-9860005	BGIOSGA025492	9667723-9668514	OsFBX233 - F-box domain-containing protein, expressed

ORF25	Os07g0270301	9963261-9965398	No homologues		Hypothetical protein
ORF26	Os07g0270800	9963285-9965579	BGIOSGA025498	9806363-9808684	Expressed protein
ORF27	Os07g0270900	9972367-9976187	BGIOSGA025499	9815737-9818831	Mak16 protein domain-containing protein, expressed
ORF28	Os07g0270950	9976525-9980344	BGIOSGA024492	9819927-9822697	Glycosyl transferase, group 1 domain-containing protein, expressed
ORF29	Os07g0271000	9982698-9988539	BGIOSGA024491	9825732-9831104	rab GDP dissociation inhibitor alpha, putative, expressed

Table S5. Polymorphic DNA markers used in heading-date QTL analysis in 76 CSSLs and the secondary F_2 (BC_5F_2) populations.

Marker	Chr.	Forward primer	Reverse primer	Start to stop
RM10018	1	ACTAGTACACCTCAACTTCACTCC	CCTTTAGTTTGCTTGTGACC	270317 to 270464
InD1	1	AGGGGAAGAAAAACCTGACC	CCGCGTGCAGATAAAGTACA	385496 to 385515
RM6470	1	ACCTTTCCCATGGTGGAATC	TTATACCTTCGACGGGAACG	1640045 to 1640234
RM3746	1	AAATGGGCTTCCTCCTCTTC	CAGCCTTGATCGGAAGTAGC	6152799 to 6152937
RM8147	1	GATTGGCTTGATCGAATTAG	CTGAGAAATTGTGTCCAATG	8452616 to 8452746
RM3627	1	GGCTACTCGAGCAAGCTCTG	ACCTACCCGTCATCCCTCTC	10308900 to 10308996
InD7	1	TGGCCCAATAGCCCATTTAT	CGAGAGCCCGAGAGAGAGA	15301708 to 15301727
RM7075	1	TATGGACTGGAGCAAACCTC	GGCACAGCACCAATGTCTC	15446661 to 15446815
RM8004	1	TTGACCAAAGGTGATTGTAAT	CTTGATGAGTTTCATGAGCA	19326401 to 19326510
RM212	1	CCACTTTCAGCTACTACCAG	CACCCATTTGTCTCTCATTATG	33381385 to 33381546
InD15	1	CAACCCCTCCAAATACCTGA	ACCGTGTTCATGCCTTTCAC	36947575 to 36947594
RM12276	1	GTCGACGGCTTCCTCAAGATTGG	TGAGACCTCTGTGAAGGCACTCG	40253681 to 40253866
RM3520	1	TGCTACTCCTCTCGCCTTTC	CACAAAAACAGTCAGCCACG	43371168 to 43371356
RM110	2	TCGAAGCCATCCACCAACGAAG	TCCGTACGCCGACGAGGTCGAG	1326951 to 1327085
InD21	2	ATAGGGTGGGTGTGCTGAAC	GCACAAAACTGCAGGTCTCC	5477492 to 5477511
RM145	2	CCGGTAGGCGCCCTGCAGTTTC	CAAGGACCCCATCCTCGGCGTC	7707108 to 7706915
RM324	2	CTGATTCCACACACTTGTGC	GATTCCACGTCAGGATCTTC	11389878 to 11389764
InD24	2	TACCTCGGCTCGGGTCAAT	CGACCAAGCGAGAAGGTACT	13013826 to 13013844
RM3443	2	AGGCCGCACATGATAGTACC	TACACGCCTGTAGCTCGTTG	14331149 to 14331247
RM2634	2	GATTGAAAATTAGAGTTTGCAC	TGCCGAGATTTAGTCAACTA	20501153 to 20501307
InD28	2	GGCTGGCTGTTGCTCATC	AAAAATCCCAACCCTGCTG	23908710 to 23908727
RM1920	2	CAAACACAGTGTTGACAGAA	GCTATTGACTTATCCGTTCA	25467438 to 25467331
RM3512	2	ATACATGCATACTCCGATCC	TCGAGTTGTGAAAGATAGGG	27320791 to 27320966

RM6424	2	AGCGAATCAGGTGACTCCAC	ACACCATCCATCTCCAGTCC	29625976 to 29625895
InD31	2	GGAAGCTTCAGCCTCACG	GGTTATACAACGGCGGATCT	31453654 to 31453671
RM208	2	TCTGCAAGCCTTGTCTGATG	TAAGTCGATCATTGTGTGGACC	35135783 to 35136068
InD36	3	TGGTTTATATTGGAACGGAGGA	GTTACATGCCCTTTCGCAGT	1711336 to 1711357
RM3392	3	GTCCAATGATTCGTTCCCAC	CTTCACCGTTCACCAATTCC	3806062 to 3806252
RM1324	3	TGTTGATCCCCTTGATAGGG	AGCAAGATCAGCTAGCTGCC	6037301 to 6037191
RM3278	3	GAGATCGATCCAAGCGAAAG	CGGACCGCAGTTTCAGAC	9316443 to 9316610
RM282	3	CTGTGTCGAAAGGCTGCAC	CAGTCCTGTGTTGCAGCAAG	12387516 to 12387607
InD51	3	CCATCTCTTTCCACGACGAT	AGTGCGGCGAACAGATAAAG	18441927 to 18441947
RM15280	3	AGTTGAAGTGTTGACCGCAATCC	GTAGGTAGAGGCTCTGGCAGTCG	18724739 to 18724758
InD55	3	AGGTCTCGTGTCGTTCATCC	TGGAGGGAGCATGTCTATCA	24128352 to 24128371
RM3199	3	TAAAAACCTCACCTCGCTGG	TAAAAACCTCACCTCGCTGG	26881158 to 26881004
RM7097	3	GGGAGGAGGAGAGGAGATTG	TTAGGCCTGCACTTTTGGAG	30422354 to 30422335
RM7000	3	CCCTTCTTTTCAACTGAATA	TTGTAACAATGAACTCGTTC	33381422 to 33381546
RM3684	3	TATTTCACCTTCCTGCCACG	GAATGAGGTGGAGGATCGAC	33741554 to 33741691
RM16335	4	AGCACGCGTGACATGAACTTCC	GAAGAGAGGCATGTGTTGTGTTGG	1699115 to 1699258
RM5633	4	GTGTAGCTGCTAGGCCGAAC	TTCCTTTCGCTACGTTGGAC	13059370 to 13059580
InD62	4	CCACGTATAAAGCAGTTTGTTAGG	CAAGTGTGGTTGTTAGCATCAAA	13198981 to 13199004
RM1205	4	ACACGTACCCCTGCTGATTC	ATAGCAGGAGCTCGTCGTTG	19635157 to 19635258
RM5979	4	TGCTGGACCTCACTGTTCTG	ACGTGGCTCAATCAGGAAAC	20584454 to 20584649
InD65	4	GCGTACAGCGAGAGGTTGAC	TCTCTTCGCCACGGAGAC	20942035 to 20942054
RM5757	4	CCTGAGACCATATGCTGCTG	GAGGGAGCATCATTAGCTGG	21416769 to 21416919
RM3839	4	AATGGGACCAGAAAGCACAC	AAAAAGAGCATGGGGGCTAC	23870630 to 23870962
RM241	4	GAGCCAAATAAGATCGCTGA	TGCAAGCAGCAGATTTAGTG	26823418 to 26823681
InD71	4	ATGTAACCCGGCCAGAGTG	CCATTAACTGGTCGGAATCG	35570900 to 35570918
RM1024	5	GCATATACCATGGGGATTGG	GGGATTGGGATAATGGTGTG	1212762 to 1213054
RM169	5	TGGCTGGCTCCGTGGGTAGCTG	TCCCGTTGCCGTTCATCCCTCC	7477346 to 7477489
RM7293	5	CCTAGGGGATCCAAGATGTC	GCACGGATCTACATACATGC	7506163 to 7506268
InD78	5	AAATTTAGGCCAGGCAGCTT	TCTCTCACACGCTTATTCATCTTT	15732223 to 15732242
InD79	5	CGTGCCGATGACAAACTTC	GAGGATCCATGTCCACCATT	18255115 to 18255133
RM3638	5	AACTCTCACAAATGGGGCAG	CCCTCTCTCTCTCTCCCCTC	19868153 to 19868320

RM6841	5	GGCCCACATGTCAGTTACAC	CCCACCAGCCTCACTTACTG	22719338 to 22719495
InD84	5	TGAGTTTCCGGTGTTCCATA	AAGGCAAAGTCGTTCAGCTT	29530779 to 29530798
InD85	6	GCAATCTAGTAAACTGTTCGAGAAA	TGGAATTTAAACATCCTCAATGC	518537 to 518561
RM190	6	CTTTGTCTATCTCAAGACAC	TTGCAGATGTTCTTCCTGATG	1764586 to 1764729
RM510	6	AACCGGATTAGTTTCTCGCC	TGAGGACGACGAGCAGATTC	2831443 to 2831635
RM5754	6	GCGTCTTTGACACACAATGG	GTGCCCATGTTGGTAGTTTG	5234299 to 5234132
RM5963	6	CAAAGGGGGTGTCCTCTATG	GTTGCTCGTCCTACATGTGC	8815797 to 8815639
InD90	6	CCTCATCCAGGGGTCATGTA	CGGTCAAGTGTCATCCAGGT	13236367 to 13236386
RM20069	6	GCGAGCGAGAGGAGAGATAGACG	CGAATTCGGCACGAGTAATAGGG	16542271 to 16542428
InD94	6	GGCATTGTAGCCAATCCAGA	AAACACACTCCCCCATGAGA	19499339 to 19499420
RM162	6	GCCAGCAAAACCAGGGATCCGG	CAAGGTCTTGTGCGGCTTGCGG	24030697 to 24030716
RM30	6	ACACTGTAGCGGCCACTG	CCTCCACTGCTCCACATCTT	24035491 to 24035615
RM3394	7	GGCATTGTAGCCAATCCAGA	AAACACACTCCCCCATGAGA	652427 to 652830
InD98	7	TGACTGTTACCCTTACGTGCAG	CGGGATGAAACAGATTCTGAG	653253 to 653274
RM5752	7	TTGCAATTAATTCGATCTCC	GCAGATCGATTCGTTAGTTC	2566137 to 2566237
RM3859	7	TTGCAGATCGGTTTCCACTG	GGTCCTGGATTCATGGTGTC	8876434 to 8876702
RM5436	7	TGAGCTGCACAAGACAGACAAGC	ACCATTTGAACAGGATGGACTGG	9075706 to 9075847
InD103	7	CCCCATGAGGCCTACACTT	AGCAGCATAATCAGATGAGACG	13976024 to 13976042
RM5875	7	TTTCCCACCAGAGGAAGATG	AAGTTCCCAAGTTGGATCCG	15997148 to 15997215
RM1132	7	ATCACCTGAGAAACATCCGG	CTCCTCCCACGTCAAGGTC	22349892 to 22350066
RM455	7	AACAACCCACCACCTGTCTC	AGAAGGAAAAGGGCTCGATC	23985575 to 23985668
InD108	7	GCCCACCTGTCATTGAGAGTA	GTTTTTGCGCTTTTGTTGCT	26591695 to 26591715
RM1364	7	AAGAAATTCAAAACACATGA	AAAACATCTACTTTGATCCA	26773305 to 26773462
RM172	7	TGCAGCTGCGCCACAGCCATAG	CAACCACGACACCGCCGTGTTG	29560613 to 29560729
RM337	8	GTAGGAAAGGAAGGGCAGAG	CGATAGATAGCTAGATGTGGCC	146952 to 147138
RM1376	8	CATGTGTGATGACTGACAGG	GGTGCTGTGATGATTCTTTC	3162523 to 3162711
RM5556	8	ATCTCCCTCCCTCTCCTCAC	TCCACACCTTCACAGTTGAC	4589506 to 4589588
RM22529	8	TGCGAGTATTTAACTCACCCATCC	CTTGCCTCACAAGATCCAAACC	5182161 to 5182326
InD113	8	TTTTAAAGCTGCGCCAAAAG	CATAACCGGTAAAGGAGTAGCC	5546152 to 5546171
RM5767	8	CTAGCAGCCACATCAAGCAG	CTCATCCTCTCCACGCTCTC	18821607 to 18821685
RM5485	8	CTTCCACAAGCTTGGCTAGG	AATGCCATCCCCTACTCATG	24072904 to 24073024

InD121	8	AACCATGAATGAATCCCTGA	TGCAACTGACATCCTGCAAT	25715398 to 25715417
RM23662	9	GAGAGGACGATGGCACTATTGG	CGAGGAACTTGATTCGCATGG	430978 to 431127
RM7212	9	ATTGTAGGAGCGCCATATGG	GAGCTGGGTAACGAGTCGAG	6600963 to 6601114
RM5526	9	TCAGCCTGGCCTCTCTTATC	ATGATCCTCCACCCACTAGC	7313032 to 7313202
InD126	9	GCCGGCCTTATCCATTTTT	GAGCGCCACTGCTTCTACTC	8640972 to 8640990
RM1328	9	GAATGGGATTAGACGATTTG	CCATGAGTGACATCAAAAGG	9206385 to 9206570
RM296	9	GCGCTGGTGGAAAATGAG	GGCATCCCTCTTTGATTCCTC	11886205 to 11886396
RM6839	9	GAACAGAGGAGGAGATCGAGAGG	CTTCTTGGGAGATGCAGAAATGG	14566026 to 14566255
InD129	9	GCGAACCGATAAAACTGCTC	AGAGGTGTATCAAAGCAATCGAG	16190620 to 16190639
RM24718	9	TGACGTGGCAAGTTGACTGTGG	TAGCCGATGGAGCCACTAGAAGG	21204919 to 21205076
InD133	10	AATTCTTATGGACGGATACGC	TCAGCATCTCGTAAGCAAAAA	504778 to 504798
InD135	10	TTTCTCCTTTCATCCACTGCT	AACGTGGAACCCTAGTCAAGAA	5456283 to 5456303
RM25152	10	ACCACCACTCTCTCCCTCAACG	CCCTCGAGGAACATATCCAAACC	7559237 to 7559375
RM311	10	TGGTAGTATAGGTACTAAACAT	TCCTATACACATACAAACATAC	9487385 to 9487518
RM6142	10	TCTTCCTCACCTGCTTCTCC	TACAGAGGCTACTACCACGACG	12873229 to 12873307
RM5620	10	TCGACTTGAAGCATCACACC	TCTGAAATGTCAAGTGGGCC	17475024 to 17474867
RM6100	10	TCCTCTACCAGTACCGCACC	GCTGGATCACAGATCATTGC	18887956 to 18888100
RM3773	10	CTGGATGAAAGGATACAACA	CACATTATCTGTCAAGGTCC	19966793 to 19966663
RM5471	10	AAGGACGGTGCAATTCTCAC	GTAGCAGCAAAAGGGAAAGG	21922317 to 21922447
RM3834	10	CTCGAGCTCCAACAAGAACC	GCTATGCTGAGCCGGAGTAG	21951156 to 21951421
RM286	11	GGCTTCATCTTTGGCGAC	CCGGATTCACGAGATAAACTC	383711 to 383945
RM26063	11	GATCCATATGCCTCTTCGATTGG	AACTCCAGCAGTGAGAGCGTAGC	2256991 to 2257112
InD144	11	TGATGAGCTCTCACTTGTTGAAA	CGTACATTGGCTTATGTGATCTG	3333209 to 3333231
RM5704	11	AACGAATGATTAAACATCTA	AAGCAGAGTCAACATATTTA	5481600 to 5481436
RM7463	11	CGAGGATCACACCAGTTTTG	TGCAGAGCCAAGAAAGGAAG	10196422 to 10196255
RM26652	11	CAATCCATTGCTGGTTGATGC	CAAGATCTCCAAGGTGCTGAGG	15031421 to 15031590
InD151	11	TGCAGTACAACACTCAGTTCAAA	CATGTTACGGTACTGGCATCA	23319353 to 23319375
RM5926	11	ATATACTGTAGGTCCATCCA	AGATAGTATAGCGTAGCAGC	28399561 to 28399855
InD156	12	TCTCAAGCATGTCAAGGCTTA	ATGAACATGCAGAGCACCAA	3028825 to 3028845
RM7003	12	GGCAGACATACAGCTTATAGGC	TGCAAATGAACCCCTCTAGC	6776293 to 6776212
InD159	12	TGGGCAACTGAATCTAACCA	GGAGATGATGATGCGGTGAT	10474558 to 10474577

RM27973	12	CCACACTGCCCAGGATTTAAGC	CTGTTCCCATCATCCAAATGACC	12266561 to 12266848
RM7018	12	CATCGTTGACCGCTGCTC	AATAAACAGCACGTGCTCCC	22162930 to 22163064
InD165	12	TCAGACACAACGTACACATCG	TCGATTGATCACTGACGGTTA	25637644 to 25637664
RM1300	12	CAGCCATGAATGTTGGCTAC	GCCATGTCCATTTATGGTGC	25965402 to 25965555

Table S6. DNA markers used in QTL analysis and fine mapping of *qHD7b*.

Marker	Chr.	Type	Forward primer sequence	Reverse primer sequence	Start to stop
InDel4373	7	InDel	AGTCTTTGATGGAAAGCTCC	TATTCCCAAAATTTTCATGG	8906561 to 8906729
InDel4477	7	InDel	TTCTTTAAGCAATCAAAGGG	CGTCCTACATGTGCAAAATA	9075693 to 9075860
InDel3	7	InDel	AATAAGTGGCTGATTGTC	ACAAGCATATGGATACAT	10152283 to 10152436
InDel13	7	SSR	TCAACACGTTTTCTTACCGGA	AACCTGTCGACGGATTCTTG	11127843 to 11127916
InDel103	7	InDel	CCCCATGAGGCCTACACTT	AGCAGCATAATCAGATGAGACG	13315866 to 13315977
RM21470	7	SSR	TCTTGCCATCACATAGCAACAGG	ACTCGGTGAGCATCCAATGTCC	14619710 to 14619870
RM21478	7	InDel	TAACACAGTTCTTCTCGCAACG	AAGTTCCCTTGTGTGATTGACC	14735918 to 14736046
RM5875	7	InDel	TTTCCCACCAGAGGAAGATG	AAGTTCCCAAGTTGGATCCG	15998890 to 15998828
InDel10	7	InDel	CTACAGAGCCACAGCCTACA	GAATGGAAAGGGAAGCGGAG	8989078 to 8989180
InDel12	7	InDel	AGAAAAACGGACAGACGGAA	ACTGGGATGGAAGAGTGGAT	8997629 to 8997797
InDel25	7	InDel	CACTCTTTATAGAACATTGTT	TAACTCTTTTTGTTATAAAATT	9020709 to 9020923
RM5436	7	SSR	TGAGCTGCACAAGACAGA-CAAGC	ACCATTTGAACAG-GATGGACTGG	9075636 to 9075829
InDel4477	7	InDel	TTCTTTAAGCAATCAAAGGG	CGTCCTACATGTGCAAAATA	9075693 to 9075860
RM5499	7	SSR	GGACGAAAGGGTATTTGATTGG	CCTCAAGGTGGTCTCCTTCTCC	9988139 to 9988332
InDel11	7	InDel	AAACGGGTTTTCAAAGCACA	CTTTGGGACAAGATCTGGCA	10127535 to 10127659
InDel3	7	InDel	AATAAGTGGCTGATTGTC	ACAAGCATATGGATACAT	10152283 to 10152436

Table S7. Markers used for sequencing, qRT-PCR, and CRISPR.

**Ghd7 sequencing primers**		**Physical location**
Ghd7-M1F	GGCTGAGCCAAACAATATTGTGTTCC	9150263
Ghd7-M1R	TAGCACACAGCCACTAAAACTTGGG	9151577
Ghd7-M2F	TCTTATGGAAATTGAACTAGAGCTTAG	9151381
Ghd7-M2R	TTGATCATGGAAATGTATGATTCAAACAT	9152081
Ghd7-M3F	ACTAGCACTTGAATCCAAATAATTATCCT	9151880
Ghd7-M3R	GTACAAGGAGAAGAGGAAGAAGAGG	9152588
Ghd7-M4F	CTCGGCATAGGCTTTTCTGGAC	9152516
Ghd7-M4R	ATGTGTATATGCCTTTTTAACCCTTAATT	9153301
Ghd7-M5F	AAAAAGGCATATACACATACTATTTCCT	9153284
Ghd7-M5R	ATCGATCAATATATACCAAGTTTGAATT	9154150
Ghd7-M6F	ATATCATCGAATTAATTAAGTCGCCAT	9153989
Ghd7-M6R	CTCGATCGAGTTTGATTTATCCG	9154846
Ghd7-M7F	CGCACAGGCCACATCCTTC	9154778
Ghd7-M7R	AACTTGCAAGCTGGTGGGA	9155590
**qRT-PCR primers**		
Os07g15770-F	GGGAGAGCTTGAACCCAAAC	
Os07g15770-R	TGCTTCTCGTAGCACCTCTT	
Ubq-qRT-F	GCTCCGTGGCGGTATCAT	
Ubq-qRT-R	CGGCAGTTGACAGCCCTAG	
***Ghd7*-CRISPR primers**		
Ghd7-CRISPR-F	AGATGATCCGTGGCAGCCGGGGCGCCGATCCCCGTTTTAGAGCTATGC	
Ghd7-CRISPR-R	GCATAGCTCTAAAACGGGGATCGGCGCCCCGGCTGCCACGGATCATCT	
